# Efficacy of interventions for posttraumatic stress disorder symptoms induced by traumatic medical events: a systematic review

**DOI:** 10.1080/17437199.2025.2526666

**Published:** 2025-07-16

**Authors:** Corinne Meinhausen, Katherine Fu, Richard D. Urbina, Tanisha Gunby, Lauren A. Perez, Patrick A. Wilson, Christina M. Luberto, Jennifer A. Sumner

**Affiliations:** a Department of Psychology, University of California, Los Angeles, CA, USA; b Edson College of Nursing and Health Innovation, Arizona State University, Tempe, AZ, USA; c Department of Psychology, California State University, Los Angeles, CA, USA; d Department of Psychiatry, Massachusetts General Hospital/Harvard Medical School, Boston, MA, USA

**Keywords:** Posttraumatic, medical trauma, cardiovascular, cancer, intervention, trauma-focused

## Abstract

Serious medical events are increasingly recognised as potential triggers for posttraumatic stress disorder (PTSD). This systematic review evaluated the efficacy of interventions for medically induced PTSD. Nine electronic databases were searched from inception to November 2023 (PROSPERO ID: CRD42024504055). Eligible studies were randomised controlled trials of interventions for adults diagnosed with, or exhibiting elevated symptoms of, PTSD related to life-threatening medical events. Risk of bias (RoB) was assessed using the Cochrane RoB 2 tool. Group differences at follow-up were assessed using independent *t*-tests for statistical significance, and Cohen’s *d* was calculated to measure effect sizes. Eleven trials (sample sizes: 17–89) met inclusion criteria, with PTSD primarily resulting from cardiovascular events (*n* = 5) or cancer (*n* = 4). Interventions included trauma-focused psychotherapies (*n* = 8; e.g., Eye Movement Desensitisation and Reprocessing [EMDR]) and others (*n* = 3; e.g., supportive therapy). Most studies reported significant posttreatment differences and large effect sizes favouring the intervention group, with EMDR and other trauma-focused psychotherapies particularly well-supported. Common limitations included small sample sizes, use of self-reported outcomes, and high dropout rates. Results highlight the efficacy of several interventions for medically induced PTSD and the need for larger trials.

## Introduction

Exposure to traumatic events, such as interpersonal violence and combat, is pervasive worldwide, with over 70% of individuals experiencing at least one trauma during their lifetime (Benjet et al., 2016). Although many individuals recover psychologically from these experiences, a significant number develop symptoms of posttraumatic stress disorder (PTSD) – the quintessential trauma-related mental disorder (American Psychiatric Association, 2013). Although research has primarily focused on PTSD after external traumas (e.g., combat), PTSD can also be precipitated by serious medical events. These experiences constitute a mounting source of trauma exposure (Mokdad et al., 2018), and medically induced PTSD represents an estimated 6.5% of PTSD cases (Sommer et al., 2018).

### Diagnostic considerations for medically induced PTSD

Medical events were first recognised as potentially traumatic experiences that could induce PTSD in the fourth edition of the *Diagnostic and Statistical Manual of Mental Disorders* (*DSM-IV* American Psychiatric Association, 1994). Since then, research has shown that acute, life-threatening medical events such as those related to cardiovascular disease (CVD) or cancer can be potent triggers for PTSD development (for reviews, see Cordova et al., 2017; Edmondson & von Känel, 2017). Meta-analytic evidence suggests that clinically significant medically induced PTSD symptoms occur in approximately 12% of patients following acute coronary syndrome (Edmondson et al., 2012), 23% following stroke (Edmondson et al., 2013), and 7% following cancer diagnosis (Abbey et al., 2015). However, updated diagnostic criteria in the fifth edition of the *DSM* (*DSM-5* American Psychiatric Association, 2013) specify that a ‘life-threatening illness or debilitating medical condition is not necessarily considered a traumatic event’ unless it ‘involve(s) sudden, catastrophic events’ (American Psychiatric Association, 2013, p. 274). As such, life-threatening medical conditions that develop gradually, like cancer, may not qualify as index traumas for PTSD diagnoses. However, elements of cancer diagnosis and treatment – such as sudden disease progression – can evoke intense feelings of fear and helplessness, mirroring key diagnostic considerations for life-threatening emergencies like sudden cardiac events (Cordova et al., 2017). Additionally, research suggests that medically induced PTSD is a valid and prevalent diagnostic presentation with important clinical implications (e.g., Sumner & Edmondson, 2018; Vilchinsky et al., 2017). For instance, there is evidence that those who develop PTSD symptoms following a cardiovascular event have poorer physical recovery and greater risk of mortality (Agarwal et al., 2019; Edmondson et al., 2012; Presciutti et al., 2020). As such, identifying and treating PTSD symptoms that develop following medical events may have clinical benefits, even amid debate over whether the inducing medical event meets current diagnostic criteria.

### PTSD treatment guidelines

Numerous clinical practice guidelines recognise trauma-focused psychotherapies, which employ cognitive, emotional, and/or behavioural strategies to aid in processing traumatic experiences (Schnurr, 2017), as first-line treatments for PTSD (Hamblen et al., 2019; US Gov Pr Office, 2023). In particular, Cognitive Processing Therapy, Prolonged Exposure (PE), Eye Movement Desensitization and Reprocessing (EMDR), and trauma-focused cognitive–behavioral therapy (CBT) are regarded as gold-standard treatments for PTSD, due to extensive evidence of their efficacy in improving PTSD symptoms with minimal adverse effects in clinical trials (Hamblen et al., 2019). A recent clinical practice guideline also cites some evidence in support of other trauma-focused psychotherapies (i.e., Written Exposure Therapy [WET] and Narrative Exposure Therapy) and mindfulness-based stress reduction for the treatment of PTSD, but more research is needed on their efficacy in a range of patient populations (US Gov Pr Office, 2023). Despite some evidence of the efficacy of pharmacological treatment for PTSD, trauma-focused psychotherapies are preferred due to their greater efficacy and lower risk of adverse effects (Hamblen et al., 2019).

### Treatment for medically induced PTSD

Despite the substantial literature on treatments for PTSD in response to external traumas, far less research has examined treatment specifically for medically induced PTSD. Additionally, PTSD induced by medical events may manifest differently than PTSD due to external traumas, potentially necessitating unique intervention approaches. These differences in PTSD symptom manifestations (e.g., hyperarousal keyed to internal vs. external cues) are outlined in the leading theoretical model of medically induced PTSD, the Enduring Somatic Threat (EST) model (Edmondson, 2014). Thus, there is a need for research examining the efficacy of treatments for medically induced PTSD to determine the applicability of traditional PTSD treatments for this population and develop clear treatment recommendations for patients and providers.

Some studies have begun to assess various psychotherapeutic interventions for medically induced PTSD, and Haerizadeh and colleagues (Haerizadeh et al., 2020) conducted a systematic review of this literature. A comprehensive literature search through November 2018 identified six randomized controlled trials (RCTs) of psychotherapy interventions for adult patients with PTSD symptoms induced by medical events, including diagnosis of a life-threatening illness. There was preliminary support for exposure-based CBT and EMDR in reducing medically induced PTSD symptoms based on three trials supporting each intervention type across a range of medical illnesses. However, the authors noted extensive variability in control conditions and PTSD measures, as well as a lack of blinding, as limitations of this literature. Despite these limitations, this review was a critical step in understanding the role of targeted psychotherapeutic interventions in addressing medically induced PTSD symptoms.

### Current study

The current systematic review aimed to collate and analyze emerging evidence on interventions designed to treat symptoms of PTSD due to medical events and build on the review by Haerizadeh and colleagues (Haerizadeh et al., 2020). Additionally, this review expanded the scope of the search to include additional medical conditions, such as COVID-19 and sepsis. By focusing on the efficacy, methodologies, and outcomes of RCTs of various therapeutic approaches, we aimed to provide a comprehensive understanding of current practices and identify gaps in the literature to enhance the care and recovery of individuals experiencing PTSD symptoms following serious medical events.

## Methods

### Search strategy

This systematic review adhered to the standards outlined by the Preferred Reporting Items for Systematic Reviews and Meta-Analyses (PRISMA) guidelines (Page et al., 2021). The protocol for this review was registered with PROSPERO, the international database of registered systematic reviews, prior to conducting the review (Registration Number:XXX). We performed a comprehensive search across nine electronic databases (Ovid MEDLINE, EMBASE, The Cochrane Library, CINAHL, PsycINFO, APA PsycNet, Google Scholar, PTSDPubs, and The PTSD Repository) from database inception through November 2023. The search strategy included a range of subject headings and free-text terms encompassing PTSD in conjunction with medical illnesses (e.g., acute condition, critical illness) or specific health conditions including CVD, cancer, human immunodeficiency virus (HIV), sepsis, and COVID-19 (for list of search terms, see Appendix). Only RCTs published in English were included. Additionally, we reviewed references from relevant reviews identified during the search to examine any further studies potentially omitted in the initial search. To identify relevant ongoing research, we searched ClinicalTrials.gov and the WHO International Clinical Trials Registry Platform.

### Eligibility criteria

To be considered for inclusion, studies needed to meet the following criteria: (1) employ an RCT methodology of a psychotherapeutic, biological, pharmacologic, or complementary/integrative intervention; and (2) target adults who experienced a life-threatening medical event, with inclusion based on PTSD diagnoses or elevated PTSD symptoms related to the medical event. Exclusion criteria included studies with: (1) children or adolescents; (2) individuals treated for PTSD following nonmedical/external trauma that resulted in medical conditions (e.g., burns from a fire or combat-related injuries); and (3) no specified PTSD inclusion criteria.

Eligible interventions could include psychotherapeutic PTSD treatments, complementary and integrative therapies, nonpharmacologic biological treatments, and pharmacologic treatments. There were no restrictions on the type of comparison group. Comparators could include other active PTSD treatments and waitlist, standard care/treatment as usual, placebo, or attention control groups. Only RCTs were considered, but feasibility studies with a randomised design and comparison group were eligible.

Two investigators from the study team independently screened each abstract and title for eligibility. The full texts of potentially eligible titles and abstracts were then reviewed independently by two study team members. In the case of disagreement, consensus was reached by a third investigator at both stages of the screening/review process.

### Data extraction

Two researchers independently extracted data from each study included in the systematic review; if a disagreement arose, consensus was reached by a third investigator. Key information collected included study title, authors, publication date, country, number of participants and demographic characteristics (e.g., age, gender), and study inclusion and exclusion criteria. We also recorded the type of PTSD intervention and comparison used, intervention duration, posttreatment dropout rates for the intervention and comparison groups, intervention modality (individual or group), and whether interventions were conducted in-person or remotely for each study. The impact of the intervention on PTSD symptoms or diagnosis (i.e., the primary outcome) at each follow-up timepoint and the PTSD assessment method were also extracted.

### Risk of bias

Our risk of bias evaluation followed the guidelines in the Cochrane Risk of Bias 2 tool for RCTs (Sterne et al., 2019). This comprehensive strategy involved assessing each study across five distinct domains: (1) the potential for bias during the randomisation process; (2) the risk of bias resulting from deviations from intended interventions; (3) the likelihood of bias due to incomplete outcome data; (4) the risk of bias from the way outcomes were measured; and (5) the possibility of bias in how results were selected for reporting. For each study, two independent reviewers assessed and assigned a risk level – low risk, some concerns, or high risk – for each domain. Based on scores in each domain, an overall risk of bias score was assigned for each study. The overall score corresponded to the highest risk of bias in any domain (Sterne et al., 2019). However, if risk of bias was judged to have ‘some concerns’ for >2 domains, the study was assigned an overall high risk of bias (Sterne et al., 2019). In the case of disagreement, consensus was reached by a third investigator.

### Statistical analysis

The literature identified in our search was neither sufficiently large nor conceptually homogeneous to support an overall meta-analysis. Indeed, there was much variability across studies with respect to the range of treatment modalities and intervention approaches, comparators, symptom measurement tools, and patient samples. However, there was one subset of three studies comparing EMDR to an active comparator that permitted a meta-analysis (Arabia et al., 2011; Capezzani et al., 2013; Carletto et al., 2016. For these studies, we conducted a random-effects meta-analysis using Hedges’ *g* as the effect of interest using the ‘meta’ package in R (version 8.0–2, Balduzzi et al., 2019). A negative Hedges’ *g* indicated greater PTSD symptom reduction in the EMDR than active comparator group, with 0.2 indicating a small effect, 0.5 indicating a medium effect, and 0.8 indicating a large effect. We quantified statistical heterogeneity using: (1) the *I*^2^ statistic (with 95% confidence intervals [CIs]), which estimates the percentage of variation across studies due to heterogeneity rather than random sampling error (Higgins et al., 2003), and (2) the prediction interval, which describes the range of the true effect of a new future study (Al Amer & Lin, 2021).

Even though the remaining studies lacked the conceptual comparability needed to conduct a meta-analysis, we nevertheless extracted relevant statistics and calculated effect sizes to support interpretation and comparison of results. Means and standard deviations (SDs) of PTSD symptom scores for the intervention and comparison groups were extracted when available; if not available, SDs were calculated using sample size and 95% CIs. We also calculated the statistical significance of the difference between the intervention and comparison groups at follow-up assessments by performing independent samples t-tests, and calculated Cohen’s *d* effect sizes when data were available (benchmarks for small, medium, and large effects are comparable to those for Hedges’ *g*). Main effects of group, Time x Group interactions, and PTSD diagnosis status at follow-up were also extracted when reported.

## Results

### Search results

**Figure 1.**
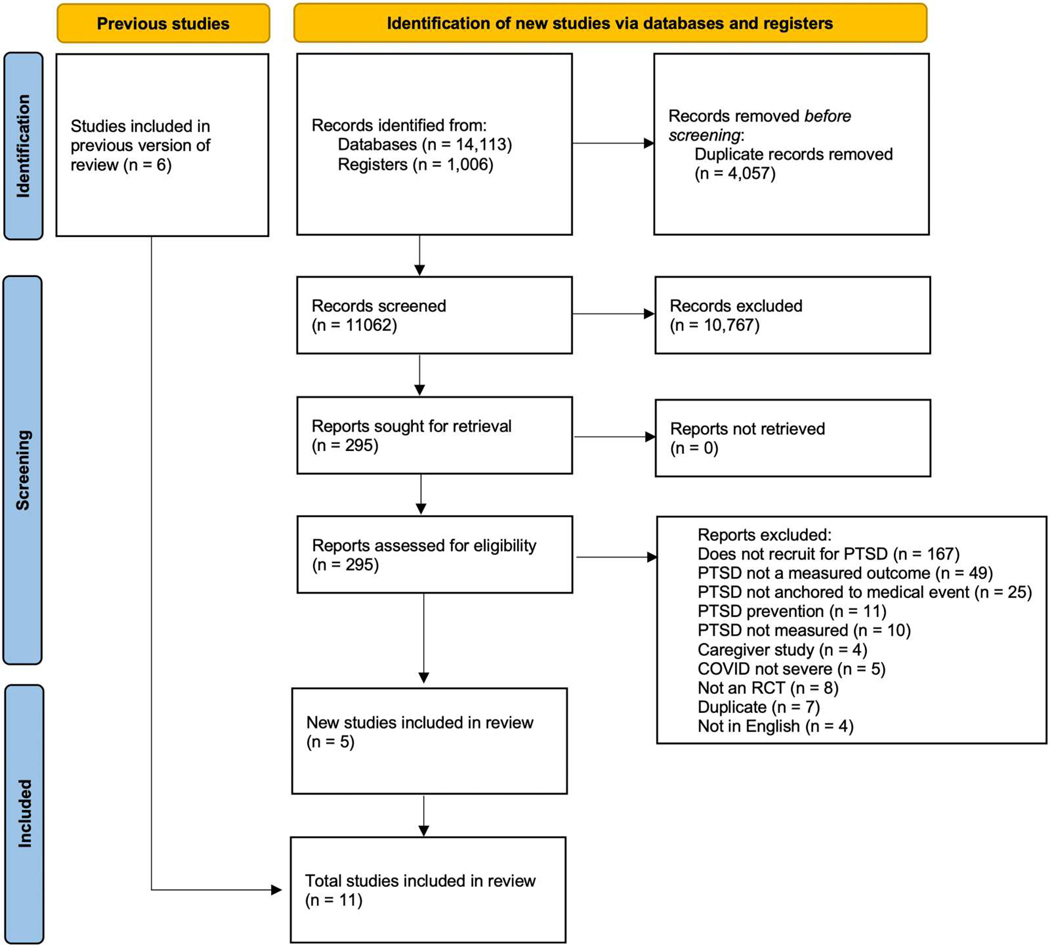
Flowchart of the systematic literature review process.

Figure 1 presents the flow diagram of the literature search and selection strategy used in the review. A total of 15,119 studies were identified, of which 4,057 duplicates were removed. Titles and abstracts of 11,062 studies were screened; of these, 295 were selected for full-text review (148 research articles, 106 clinical trial registries, 29 conference abstracts, 12 study protocols). Of the 148 research articles selected for full-text review, 138 were excluded because they did not meet eligibility criteria (see Figure 1 for exclusion reasons). Eleven studies were ultimately deemed eligible for inclusion in the systematic review: five new/previously unreviewed studies identified in our search and six studies included in the previous systematic review (Haerizadeh et al., 2020). Of the six from the prior review, five were identified in our search; one study, which was not identified in our search due to updated search criteria, was also considered relevant for inclusion in our review.

### Study characteristics

Of the 11 studies included in this review, 8 examined trauma-focused psychotherapies, including EMDR (*n* = 4), CBT (*n* = 2), PE (*n* = 1), and WET (*n* = 1). The remaining studies examined a non-trauma-focused CBT intervention, repetitive transcranial magnetic stimulation (rTMS), and a supportive therapy intervention. Most interventions were administered in person (*n* = 8), but 27% (*n* = 3) were delivered virtually. Comparison groups included inactive control groups (*n* = 4; e.g., waitlist control, weekly monitoring), active control groups (*n* = 5; e.g., health education, relaxation therapy, sham rTMS treatment), and active PTSD treatments (*n* = 2; CBT, imaginal exposure). Sample sizes ranged from 17 to 89 participants. Most studies examined PTSD symptoms that developed following either cardiovascular events (*n* = 5; e.g., stroke, myocardial infarction) or cancer diagnoses (*n* = 4; e.g., breast cancer, hematologic and lymphoid cancers). Two additional studies examined PTSD symptoms that developed in response to multiple sclerosis (MS) and HIV. Symptoms and diagnoses of medically induced PTSD were assessed using self-report (*n* = 5) or clinician-determined diagnosis (*n* = 3). Additionally, three studies screened for PTSD symptoms initially with self-report measures and confirmed with clinician-determined diagnosis. Table 1 provides a detailed summary of the characteristics of the 11 studies in this review.

### Risk of bias assessment

**Figure 2.**
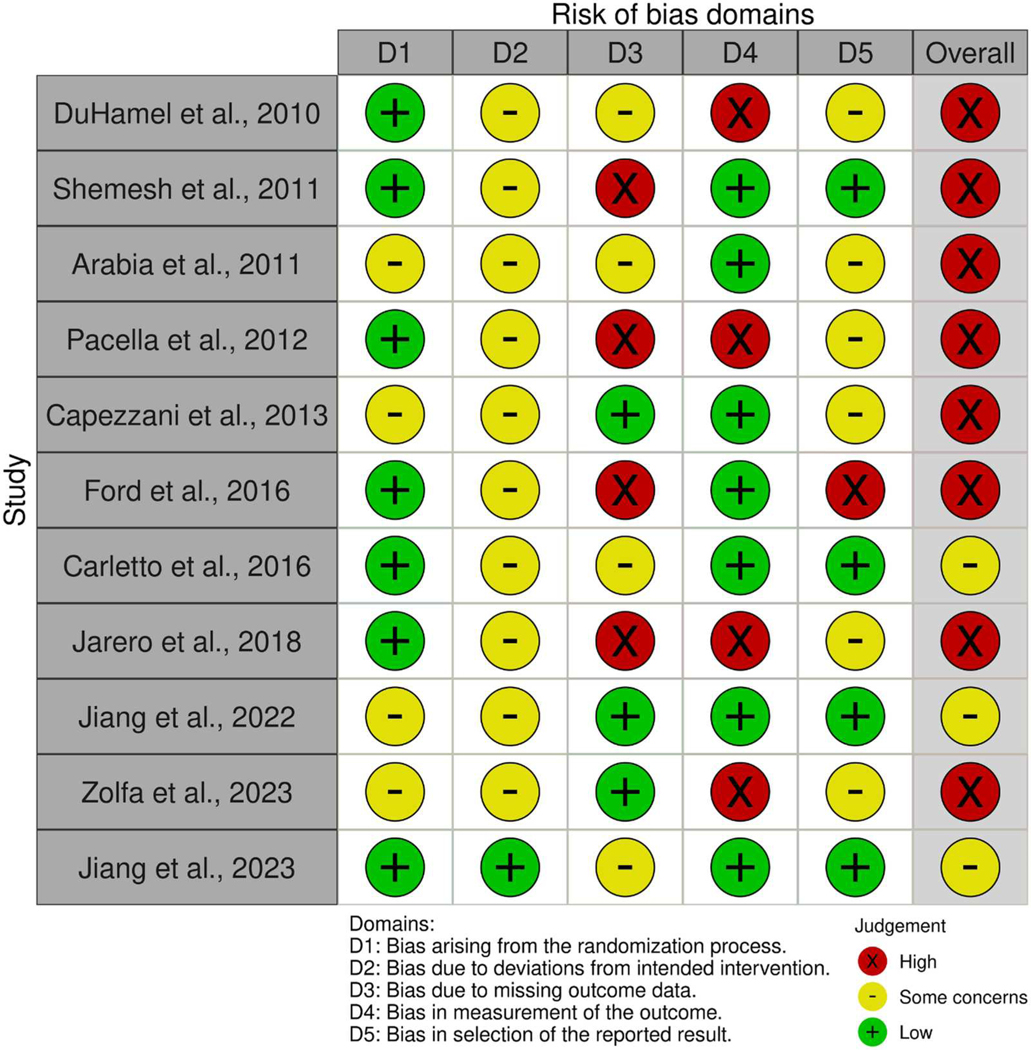
Risk of bias of included studies assessed with the Revised Cochrane Risk of Bias tool for randomised trials (RoB 2).

Multiple potential sources of bias were identified in the included studies, with 8 of the 11 studies determined to have an overall high risk of bias and 3 considered to have some risk of bias (see Figure 2 for results). The most common sources of bias stemmed from (1) the inability to blind participants and providers to group assignments due to the nature of the interventions, which were inherently distinguishable to those receiving or administering them; (2) deviations from intended interventions due to high dropout (especially in treatment groups) and a lack of information regarding whether deviations occurred; (3) the use of inactive control groups; and (4) lack of available clinical trial registries or study preregistrations.

### Study descriptions and outcomes

Table 2 presents a detailed description of the outcome results including dropout rates posttreatment and group comparisons on PTSD symptom outcomes.

### Cardiovascular-induced PTSD treatments

Five studies examined interventions for PTSD symptoms induced by cardiovascular illness. The study by Shemesh et al. (2011) evaluated the safety and efficacy of 3–5 sessions of trauma-focused CBT with imaginal exposure (*n* = 30) compared to 1–3 educational sessions on medication adherence (*n* = 30) in patients diagnosed with PTSD in response to various cardiovascular conditions. Given the focus on assessing safety, the CBT intervention did not include homework or *in vivo* exposure and was provided in a medical setting over fewer sessions than typically administered in a CBT intervention (3–5 vs. 8–15). Additionally, blood pressure and pulse were measured during administration of CBT and patient education sessions. The study reported no significant differences in cardiac measures across the two groups, and no significant adverse health events during or immediately after the intervention. Therefore, the authors deemed the exposure-based CBT intervention safe for individuals with cardiac conditions. This study did not include individual outcome data, so no effect size could be calculated. However, the authors reported that the CBT group showed larger decreases in PTSD symptoms from baseline to 2-month follow-up than those in the educational group, although this difference was not significant. Dropout was higher in the CBT group (*n* = 7, 25%) than in the education group (*n* = 2, 7%) (Table 2).

The study conducted by Arabia et al. (2011) investigated the efficacy of EMDR compared to imaginal exposure in individuals undergoing cardiac rehabilitation with elevated symptoms of PTSD due to a life-threatening cardiac event. Individuals in both EMDR (*n* = 21) and imaginal exposure (*n* = 21) received two preparatory sessions in which the treatment rationale was provided and information was gathered, followed by 45-min twice-weekly therapy sessions delivered over 4 weeks. Although both groups had significant decreases in PTSD scores from pretreatment to posttreatment and from posttreatment to 6-month follow-up, individuals in the EMDR group had significantly lower PTSD symptoms compared to the imaginal exposure group at both follow-up assessments (Table 2). There was a large effect size in favour of the EMDR group at posttreatment (Cohen’s *d* = 1.05). This study also performed a subgroup analysis in individuals with symptom scores above the clinical cutoff for probable PTSD diagnosis (Impact of Events Scale-revised [IES-R] scores ≥33; *n* = 19, 45%). Although both groups had significantly decreased PTSD scores from baseline, EMDR was associated with significantly lower PTSD symptoms compared to imaginal exposure at both follow-up assessments. The posttreatment Cohen’s *d* (1.75) was also larger for the subset of individuals with elevated IES-R scores, indicating that treatment effects of EMDR over imaginal exposure were larger in those with elevated PTSD symptoms. There was no dropout in either condition at posttreatment (Table 2).

The study by Ford et al. (2016) examined data from an RCT of patients with implantable cardioverter defibrillators (ICDs) who received 8 weekly telephone sessions of a non-trauma-focused, ICD-specific CBT intervention compared to usual cardiac care. The CBT treatment included a patient education book and stress management procedures. Although the initial RCT included individuals regardless of PTSD symptoms at baseline (Irvine et al., 2011), Ford and colleagues performed their analysis on the 31 individuals with clinically significant PTSD symptoms (mean IES-R item score ≥1.5) at baseline. The CBT group (*n* = 12) had significantly greater reductions in PTSD symptoms than those in the usual care group (*n* = 19) from baseline to 12-month follow-up, as evidenced by a significant Time x Treatment Group interaction (*F*(1, 29) = 5.95, *p* = .021, *η*^2^ = 0.17). Analysis of mean scores at 12-month follow-up showed a large effect size favouring the treatment group over usual care (Cohen’s *d* = 0.93). Dropout rates in the high PTSD symptom subgroup were not reported. However, at posttreatment in the overall sample from the parent study, 17 participants were lost to follow-up (18%) in the CBT group compared to 8 participants lost to follow-up (8%) in the usual care group (Irvine et al., 2011).

Two studies by Jiang and colleagues (2022, 2023) investigated interventions for stroke-induced PTSD symptoms. In the first study (Jiang et al., 2022), patients diagnosed with stroke-induced PTSD were randomised to a supportive therapy intervention (*n* = 9) or routine monthly health education (*n* = 9). Supportive therapy included 12 remote video sessions over 3 months, where a counselor listened to patients’ stroke experiences, set treatment goals, and encouraged family involvement in recovery. Individuals in the supportive therapy group had significantly lower PTSD symptoms than those in the control group at 1-month posttreatment; this corresponded to a large effect size (Cohen’s *d* = 1.63; Table 2). Additionally, an analysis of covariance revealed that participants in the supportive therapy group exhibited significantly greater reductions in PTSD symptoms than those in the control group (*F*(1,14) = 10.29, *p* = .01, *η*^2^ = 0.42). Significantly more individuals in the health education group (88.9%) than in the supportive therapy group (25.0%) also still met diagnostic criteria for PTSD at 6-month follow-up (*p* = .02). Dropout was minimal; one individual was excluded from the supportive therapy intervention due to nonadherence (Table 2).

The second study by Jiang et al. (2023) compared the efficacy of rTMS with brief exposure (TMS + BE, *n* = 19), rTMS alone (TMS, *n* = 18), and a sham treatment group (*n* = 20) in individuals diagnosed with stroke-induced PTSD. All groups received 10 treatment sessions over the 2-week treatment period. The rTMS in the TMS + BE and TMS groups was applied to the right dorsolateral prefrontal cortex, using low-frequency stimulation, and the TMS + BE condition also included script-driven imagery of the stroke event as the brief exposure component. Stroke-related PTSD symptoms were measured at baseline, after each week of treatment, and at 3-month follow-up. Although outcome data for groups were not reported, individuals had significantly greater symptom reduction in the TMS and TMS + BE than sham treatment groups at the end of the first treatment week, with the TMS + BE group exhibiting greater PTSD symptom improvement than the TMS group. However, at 3-month follow-up, stroke related-PTSD symptoms were significantly lower in the TMS + BE and TMS groups compared to the sham treatment group. There was no significant difference in PTSD symptoms between the TMS + BE and TMS groups. Additionally, the percentages of individuals meeting PTSD diagnostic criteria for PTSD at 3-month follow-up were lower in the TMS + BE (38.9%) and TMS (25.0%) groups than the sham treatment group (68.8%). Dropout in this study was relatively low; five participants withdrew from the trial during treatment due to uncomfortable feelings caused by rTMS (two from TMS + BE, two from TMS, one from sham treatment).

### Cancer-related PTSD treatments

Four studies examined interventions for individuals with PTSD related to cancer diagnosis and treatment. The study by DuHamel et al. (2010) evaluated a telephone-administered exposure-based CBT intervention for individuals (*n* = 52) compared to an assessment-only control group (*n* = 37) in individuals experiencing elevated PTSD symptoms related to a recent hematopoietic stem cell transplantation. The CBT intervention involved 10 60–90 min sessions over 10–16 weeks that included guided exposures to trauma-related cues, relaxation training, and addressing maladaptive emotional reactions to illness and transplantation. Follow-up measures were obtained at 6-, 9-, and 12-month follow-ups. There was a significant main effect of treatment group; individuals who received CBT had fewer PTSD symptoms compared to those in the assessment-only group across all assessments (*t*(80) = 2.37, *p* = .02). There was a medium effect size favouring CBT over assessment-only at 6-month follow-up (Cohen’s *d* = 0.51). Additionally, the log odds of meeting PTSD diagnostic criteria were significantly lower in the CBT vs. control group at 12-month follow-up (*p* = .04). Dropout was low and <10% in both groups (Table 2).

A pilot study conducted by Capezzani and colleagues (2013) compared the efficacy of EMDR to CBT in individuals diagnosed with cancer-related PTSD. Patients with various cancers in the follow-up phase of their disease were randomly assigned to EMDR (*n* = 11) or CBT (*n* = 10). The EMDR condition consisted of psychoeducation and reprocessing and integrating traumatic cancer-related memories, whereas the CBT condition varied depending on the PTSD symptoms reported by the patients and could involve guided visualisation, gradual or prolonged exposure, relaxation and attention shifting techniques, and cognitive restructuring. PTSD symptoms at posttreatment were significantly lower in the EMDR than CBT group; this difference corresponded to a large effect size (Cohen’s *d* = 1.61; Table 2). A significant Time x Treatment Group interaction (*F*(1,19) = 14.04, *p* = .001) was also found, such that those in the EMDR condition had greater improvements in PTSD symptoms over time than those in the CBT condition. Individuals in the EMDR group also had significant reductions in PTSD symptoms from pre- to posttreatment, whereas individuals in the CBT group did not. Additionally, only 9.1% of patients (*n* = 1) in the EMDR group still met diagnostic criteria for PTSD at posttreatment, compared to 90.0% of patients (*n* = 9) who received CBT. No dropout was reported across either condition.

A study by Jarero et al. (2018) provided an adapted EMDR protocol for the treatment of ongoing traumatic stress in adult women with heightened PTSD symptoms due to cancer diagnosis and treatment. This protocol – EMDR Integrative Group Treatment Protocol adapted for ongoing traumatic stress (EMDR-IGTP-OTS) – acknowledges that trauma exposures such as medical events may be ongoing and targets multiple traumatic events from pre-diagnosis to the present and potentially into the future. The EMDR group (*n* = 35) received 6 EMDR sessions, provided in a group format over 2 consecutive days; 30 individuals were randomised to a no-treatment control group. PTSD symptoms at posttreatment were significantly lower in the EMDR than control group; this corresponded to a large effect size (Cohen’s *d* = 1.65; Table 2). EMDR also led to greater improvements in PTSD symptoms over time, as indicated by a significant Time x Treatment Group interaction (*F*(2,118) = 30.66, *p* < .001, *η*^2^ = .342). At posttreatment, no dropout was reported from the EMDR-IGTP-OTS group; 13% (*n* = 4) dropout was reported from the no-treatment control group.

Zolfa and colleagues (2023) tested WET for treating clinically significant cancer-related PTSD symptoms in Iranian women with breast cancer. Participants were randomly assigned to five weekly sessions of WET (*n* = 23) or a standard care control group (*n* = 23). Participants in WET were instructed to write for 30 min about their breast cancer experience, including the thoughts and feelings they experienced. At the beginning of each subsequent session, therapists provided feedback and offered suggestions about adhering to the treatment protocol. At posttreatment, the WET group had significantly lower PTSD symptoms than the control group; this corresponded to a very large effect size (Cohen’s *d* = 4.11). Symptoms of PTSD remained significantly lower in the WET vs. control group at 3-month follow-up (Table 2). No dropouts were reported from either group at posttreatment.

### PTSD from other life-threatening medical conditions

Two studies examined PTSD that developed in response to other serious medical conditions. Pacella et al. (2012) examined PE for individuals with a PTSD diagnosis due to HIV-related and non-HIV-related trauma (here, we focus on results for PTSD symptoms related to HIV, in line with the aims of the review). Thirty-four participants were assigned to 10 90–120 min sessions of a standardised PE protocol delivered over 5 weeks consisting of psychoeducation and imaginal/in-vivo exposure. The control group (*n* = 24) received usual care with weekly symptom monitoring. A significant Time x Treatment Group interaction was observed, with participants in the PE group exhibiting greater decreases in HIV-related PTSD symptoms than those in the control group (*F*(2,128) = 4.86, *p* < .001, *η*^2^ = 0.14); there was a large effect size in favour of PE at follow-up (Cohen’s *d* = 1.90; Table 2). However, dropout in the treatment group was 32% (*n* = 11) at posttreatment, compared to 0% in the control group.

A study by Carletto et al. (2016) compared EMDR (*n* = 25) and relaxation therapy (*n* = 25) for PTSD related to progressive or relapsing-remitting MS. Participants in both groups completed two initial sessions to assess trauma histories, followed by 10 60-min individual treatment sessions of EMDR or relaxation therapy over 12–15 weeks. EMDR sessions consisted of stabilisation through visualisation, followed by eye movement tracking during recall of illness-related traumatic images, whereas relaxation therapy included visualisation, breathing, and progressive muscle relaxation techniques. PTSD symptoms in both groups were significantly reduced from baseline to 6-month follow-up, with no significant posttreatment differences observed between groups (Cohen’s *d* = 0.02). However, at 6-month follow-up, significantly more patients in the EMDR group (*n* = 20, 100%) than in the relaxation group (*n* = 17, 77.3%) no longer met diagnostic criteria for PTSD (*p* = .049). Dropout was slightly higher in the EMDR (*n* = 5, 20%) than relaxation therapy group (*n* = 3, 12%) (Table 2).

### EMDR vs. active comparator meta-analysis

For the three studies of EMDR vs. an active comparator (Arabia et al., 2011; Capezzani et al., 2013; Carletto et al., 2016), we performed a random-effects meta-analysis using the Hartung-Knapp method in the ‘meta’ package in R (version 8.0–2, Balduzzi et al., 2019). This analysis resulted in a large pooled effect size favouring EMDR, however this effect did not reach the threshold of statistical significance (Hedges’ *g* = −0.81, 95% CI: −2.72,1.10, *p* = .211). Between-study heterogeneity was large (*I*^2^ = 76.6%, 95% CI: [23.3%; 92.8%]). Further, the prediction interval ranged from −4.26 to 2.64, reflecting a wide range for the true effect of a new future study.

## Discussion

Research on medically induced PTSD has grown in recent years (McBain & Cordova, 2024), and this trend has been mirrored in the intervention literature. As highlighted in this systematic review, the efficacy of various interventions for PTSD induced by a life-threatening medical event has been increasingly examined across diverse patient populations, including those with CVD, cancer, HIV, and MS – with the number of RCTs nearly doubling in the 5 years since the previous systematic review (Haerizadeh et al., 2020). Several interventions examined in this review have shown preliminary efficacy in reducing PTSD symptoms due to a range of medical traumas, with EMDR and other trauma-focused exposure interventions emerging as particularly well-supported when compared to various conditions across multiple studies. This finding aligns with the broader PTSD treatment literature, where trauma-focused psychotherapies are consistently found to be the most efficacious (Hamblen et al., 2019). The scope of interventions examined has expanded since the preceding review and encompasses novel interventions for this population, such as WET and rTMS, both of which emerged as promising treatments for medically induced PTSD. Of note, both interventions require relatively low time commitments, which may support treatment engagement (and was mirrored by low dropout). Additionally, a sizable number of the interventions in this review were delivered remotely, which may have increased their accessibility. These trends reflect promising developments in the intervention literature for medically induced PTSD and merit further consideration to understand how these treatment delivery features may impact mental and physical health outcomes.

Most of the interventions reviewed were gold-standard, trauma-focused PTSD interventions (i.e., EMDR, trauma-focused CBT, and PE). These treatments often evidenced benefits in terms of symptom reduction compared to other treatments or no-treatment control groups, and most resulted in medium to large effect sizes favouring the intervention group. Two exceptions were a study of trauma-focused CBT (Shemesh et al., 2011) and a study of EMDR (Carletto et al., 2016); these studies failed to detect statistically significant findings and had small effect estimates (if available). Overall, outcomes for CBT were generally less promising than for EMDR, and a direct comparison of EMDR and CBT indicated a large effect favouring EMDR (Cohen’s *d* = 1.61) (Capezzani et al., 2013). The focused meta-analysis of the three EMDR trials with active comparators (Arabia et al., 2011; Capezzani et al., 2013; Carletto et al., 2016) offers some evidence of the efficacy of EMDR over other active treatments, but results were not conclusive. Although the pooled effect size was large and favoured EMDR (Hedges’ *g* = 0.81), it was not statistically significant. Furthermore, quantitative estimates of heterogeneity were large, mirroring variability in the studies (e.g., the different active comparators used). Taken together, these results suggest that although EMDR is likely a promising intervention for medically induced PTSD, there is insufficient evidence to support the superiority of EMDR over other interventions. More research is thus needed to identify the most efficacious intervention approach for this population. Furthermore, greater comparability across studies in the future will help to support more comprehensive meta-analyses.

Two of the 11 interventions examined were adapted to address unique characteristics of medically induced PTSD. The 2-day EMDR-IGTP-OTS intervention investigated by Jarero and colleagues (2018) was designed to address the ongoing nature of a medical traumatic event, aligning with a key component of the EST model (Edmondson, 2014). The effect size for this intervention was large (Cohen’s *d* = 1.55) and comparable to several other RCTs of traditionally administered EMDR provided over a longer period. However, the EMDR-IGTP-OTS was the only RCT of EMDR that did not use some form of active control group, which limits comparison of effect sizes across these studies. Thus, more research is needed to determine whether tailoring treatments for these patients yields improved outcomes. The other adapted intervention by Shemesh and colleagues (2011) compared a shortened CBT protocol without homework or *in vivo* exposure to medication education and found no significant difference in PTSD symptom reduction between the groups. There were also no adverse physiological reactions during treatment, thus providing initial evidence of the safety of exposure-based interventions for individuals with cardiac-related conditions. The addition of health-related biomarker outcomes, such as those measured by Shemesh et al., along with other physiological measures (e.g., sleep disturbance, autonomic reactivity), may broaden the scope of future research. These outcomes are particularly relevant for individuals with PTSD symptoms after CVD, given their association with both PTSD symptom manifestations and CVD risk (Meinhausen et al., 2022; Pole, 2007; Sumner et al., 2023).

Relatively high posttreatment dropout rates (0% to 32%) were observed in many of the studies in this review, potentially compromising the integrity of the findings. The highest dropout was found in the intervention conditions in RCTs of CBT and PE (23–32% Pacella et al., 2012; Shemesh et al., 2011). The higher dropout rates in treatment groups may also be a result of inactive control groups, used by four of the included studies, which limited the internal validity of the results. Attrition is common in RCTs, and meta-analytic evidence suggests an 18% dropout rate in PTSD treatment trials (Imel et al., 2013). Furthermore, dropout increased with the number of treatment sessions (Imel et al., 2013). In the current review, 2 of the 3 studies with intervention dropout rates >18% had some of the greatest treatment time burdens (Carletto et al., 2016; Pacella et al., 2012). Further research is needed to identify and address the factors that contribute to high treatment dropout in this population.

Several additional limitations merit consideration when interpreting findings and designing future RCTs. Many studies were characterised by small total sample sizes and even smaller subgroup analyses of individuals with clinically significant PTSD symptoms. As evidence grows for promising interventions, larger studies with longer follow-ups are needed to strengthen the research literature. Blinding was also an issue in many studies, given the nature of receiving a therapeutic intervention. Additionally, all studies utilised self-reported PTSD symptoms as the primary outcome, which may introduce bias. While these measures are validated, widely used, and easy to administer, the incorporation of more stringent clinician-determined diagnoses (used as an additional outcome in five of the included studies) could improve the rigour of future research (Edmondson et al., 2012). Despite these limitations, unique strengths of this systematic review include the rigorous design inclusion criteria (all RCTs) and demonstration of symptom reduction for several trauma-focused psychotherapy interventions across diverse patient populations and treatment modalities. The inclusion of various medical conditions further underscores the potential generalizability of these approaches.

Future studies should include RCTs examining the effects of these interventions in individuals with heightened PTSD symptoms due to other life-threatening medical illnesses (e.g., severe COVID-19; sepsis) which, at the time of this review, have not yet been conducted. The implementation of rigorous comparison conditions, such as active control groups, in future research is also recommended to ensure that observed outcomes are a result of the intervention components. Given the strong somatic component of medically induced PTSD (Edmondson, 2014), interventions that simultaneously treat co-occurring physical symptoms (e.g., chronic pain) and/or hypersensitivity to physical sensations (e.g., overattentiveness to increased heart rate) – such as mindfulness-based treatments (Chang et al., 2023; US Gov Pr Office, 2023) and interoceptive exposure (Farris et al., 2024; Farris et al., 2025) – warrant further research. Such approaches may help individuals better tolerate distress associated with internal somatic cues. Additionally, it is of interest for future studies to examine changes in hypothesised mechanisms (e.g., reactivity to trauma reminders), as has been examined in individuals with PTSD due to nonmedical trauma (Wangelin & Tuerk, 2015).

The integration of PTSD treatment in primary care settings may be especially promising for those with medically induced PTSD. Numerous care models, such as Integrated Behavioral Health Care (Blount, 1998) and the Collaborative Care Model (CoCM Archer et al., 2012), have successfully embedded psychological and behavioural services into primary care settings for the treatment of anxiety and depression. However, research examining these models for the treatment of PTSD, defined broadly, is limited, and findings are mixed. For example, of the four studies that have examined CoCM for the treatment of PTSD (Engel et al., 2016; Fortney et al., 2015; Meredith et al., 2016; Schnurr et al., 2013), only two demonstrated improved PTSD outcomes over usual care (Engel et al., 2016; Fortney et al., 2015). These findings suggest that applying these models to the treatment of PTSD treatment may present unique challenges. Research has demonstrated that feasibility is paramount to the success of an integrated psychological intervention, with treatments ideally ranging from four to six 30-min sessions (Robinson et al., 2005). Thus, most gold-standard PTSD treatments may be too time-intensive for primary care environments. Brief versions of gold-standard PTSD treatments adapted for primary care settings, such as Prolonged Exposure for Primary Care (PE-PC Rauch et al., 2017; Rauch et al., 2023) offer promising avenues for the integration of treatment for medically induced PTSD treatments into primary care. Additionally, an RCT of WET (an intervention examined by a study in this systematic review) is currently underway in a primary care setting (Meredith et al., 2024).

## Conclusions

The increase in RCTs in this research area and diversity of interventions examined since the previous systematic review by Haerizadeh and colleagues (Haerizadeh et al., 2020) suggest a growing recognition of the need for accessible, efficacious treatments for patients with medically induced PTSD. Based on the findings of this review, both gold-standard trauma-focused psychotherapies, such as EMDR, and alternatives, such as WET, EMDR-IGTP-OTS, and rTMS, show preliminary efficacy in the treatment of medically induced PTSD symptoms. These latter treatments also have the advantage of being more time- and cost-efficient than traditional treatments, potentially making them more accessible and feasible – particularly in medical settings. Comprehensive research aimed at refining and modifying these promising interventions to best treat the unique manifestations of medically induced PTSD is needed, as the optimal treatment of PTSD symptoms induced by medical events – and its impact on psychological and physical health – remains unclear.

## Supplementary Material

Supp 1

Supp 2

Supp 3

## Figures and Tables

**Table 1.** Characteristics of included studies (*N* = 11).

First author, Year	% Female	Mean age (*SD*)	Medical condition	PTSD inclusion at baseline	Intervention (*n*)	Session frequency/Duration	Comparison (*n*)	Delivery mode

DuHamel et al., 2010	50.6	51.01 (11.81)	Stem-cell transplantation	(1) PTSD diagnosis (PCL-C); (2) total PTSD symptoms ≥ 1 SD than the mean (PCL-C); or (3) scores exceeding cutoff for ≥ 1 PTSD symptom cluster (PCL-C)	CBT with trauma focus (52)	10 sessions/10–16 weeks	Assessment only (37)	Virtual
Shemesh et al., 2011	33.3	57.14 (10.99)	Cardiac event	Diagnosis of PTSD (SCID)	CBT with trauma focus (30)	3–5 sessions/NR	1–3 sessions of medication education (30)	In-person
Arabia et al., 2011	33.3	63.48 (10.32)	Cardiac event	Elevated PTSD symptoms (IES-R ≥ 22)	EMDR (21)	Twice per week/4 weeks	8 imaginal exposure sessions (21)	In-person
Pacella et al., 2012	36.9	46 (NR)	HIV	Elevated PTSD symptoms (PDS), PTSD confirmed (SCID)	PE (34)	Twice per week/5 weeks	Weekly monitoring (24)	In-person
Capezzani et al., 2013	90.5	51.72 (8.00)	Cancer (various)	Diagnosis of PTSD (CAPS)	EMDR (11)	Once per week/8 weeks	8 weekly CBT sessions (10)	In-person
Ford et al., 2016	NR	NR	ICD placement for arrhythmia	Elevated PTSD symptoms (IES-R mean item score ≥ 1.5)	CBT (12)	Once per week/8 weeks	Usual cardiac care (19)	Virtual
Carletto et al., 2016 *	81	40.11 (10.82)	Multiple sclerosis	Elevated PTSD symptoms (IES-R ≥ 33), PTSD confirmed (SCID)	EMDR (25)	10 sessions/12–15 weeks	Relaxation therapy (25)	In-person
Jarero et al., 2018	100	47.02 (NR)	Cancer (various)	Elevated PTSD symptoms (PCL-5 ≥ 30)	EMDR (35)	3 times per day/2 days	Waitlist control (30)	In-person
Jiang et al., 2022	29.4	56.35 (11.87)	Stroke	Diagnosis of PTSD (CAPS-5)	Supportive therapy (9)	Once per week/ 12 weeks	Monthly health education (9)	Virtual
Zolfa et al., 2023	100	41.04 (9.38)	Cancer (breast)	Elevated PTSD symptoms (PCL-5 ≥ 33)	WET (23)	Once per week/5 weeks	Assessment only (23)	In-person
Jiang et al., 2023	36.8	55.62 (8.41)	Stroke	Diagnosis of PTSD (CAPS-5)	TMS (18) TMS + BE (19)	5 days per week/2 weeks	Sham TMS (20)	In-person

Note. CAPS: Clinician-Administered PTSD Scale; CAPS-5: Clinician-Administered PTSD Scale for DSM-5; CBT: Cognitive Behavioral Therapy; EMDR: Eye Movement Desensitization and Reprocessing; HIV: human immunodeficiency virus; ICD: Implantable Cardioverter Defibrillator; IES-R: Impact of Event Scale-Revised; NR: not reported; PCL-5: PTSD Checklist for DSM-5; PCL-C: PTSD Checklist-Civilian Version; PDS: Posttraumatic Diagnostic Scale; PE: Prolonged Exposure; PTSD: posttraumatic stress disorder; TMS: Repetitive Transcranial Magnetic Stimulation; TMS + BE: Repetitive Transcranial Magnetic Stimulation plus Brief Exposure; SCID: Structured Clinical Interview for DSM; SD: standard deviation; WET: Written Exposure Therapy

* Study included in previous review [19] and was incorporated here to provide a comprehensive description of the existing literature. However, it was not identified in our search, as terms related to multiple sclerosis were not included in our search strategy given that it is unlikely to meet the diagnostic criteria for PTSD under *DSM-5*.

**Table 2.** Study results (*N* = 11) including dropout rates and group comparisons on PTSD symptom outcomes.

First author, Year	Treatment/Comparison	Posttreatment dropout % (*n*) Treatment	Posttreatment dropout % (*n*) Comparison	Primary outcome (Measure)	Baseline mean (*SD*) Treatment	Baseline mean (*SD*) Comparison	Follow-up assessment	Follow-up mean (*SD*) Treatment	Follow-up mean (*SD*) Comparison	*p*-value^a^	Cohen’s *d* Effect Size^b^

DuHamel et al., 2010	CBT with trauma focus/Assessment only	9.6 (5)	8.1 (3)	PTSD symptoms (PCL-C)	32.05 (12.39)	33.97 (11.36)	6-mon 9-mon 12-mon	25.38 (12.57) 24.63 (12.09) 24.00 (15.00)	32.05 (13.97) 31.99 (13.10) 30.89 (13.07)	.030* .012* .031*	0.51 0.59 0.48
Shemesh et al., 2011	CBT with trauma focus/Medication education	23.3 (7)	6.7 (2)	PTSD symptoms (IES)	NR	NR	2-mon	NR	NR	>.05	NA
Arabia et al., 2011	EMDR/Imaginal exposure	0 (0)	0 (0)	PTSD symptoms (IES-R)	Total sample: 33.62 (7.21) IES-R > 33: 40.00 (6.65)	Total sample: 32.29 (7.21) IES-R > 33: 40.88 (6.27)	PT 6-mon PT 6-mon	12.10 (7.19) 7.95 (7.86) 13.73 (6.66) 8.03 (7.52)	19.67 (7.21) 13.64 (7.57) 25.13 (6.27) 17.67 (6.60)	.004 .022 .001 .009	1.05 0.74 1.75 1.35
Pacella et al., 2012	PE/Weekly monitoring	32.3 (11)	0 (0)	PTSD symptoms (PSS-I)	25.30 (10.26)	26.00 (9.62)	PT 3-mon	8.30 (4.83) 7.32 (6.74)	24.13 (10.66) 20.46 (8.94)	<.001* <.001*	1.90 1.65
Capezzani et al., 2013	EMDR/CBT	0 (0)	0 (0)	PTSD symptoms (IES-R)	50.91 (9.45)	54.70 (10.62)	PT	20.55 (17.85)	46.60 (14.13)	.001*	1.61
Ford et al., 2016	CBT/Usual cardiac care	NR	NR	PTSD symptoms (IES-R, mean item)	2.23 (0.70)	2.08 (0.39)	12-mon	0.98 (0.53)	1.49 (0.56)	.016*	0.93
Carletto et al., 2016	EMDR/Relaxation therapy	20 (5)	12 (3)	PTSD symptoms (IES-R)	53.05 (12.87)	51.36 (9.58)	PT 6-mon	28.25 (18.28) NR	28.68 (19.39) NR	>.05 >.05	.02 NA
Jarero et al., 2018	EMDR/Waitlist control	0 (0)	13.3 (4)	PTSD symptoms (PCL-5)	44.89 (10.26)	43.85 (11.19)	PT 3-mon	20.51 (8.80) 17.89 (11.67)	37.19 (12.89) 35.09 (9.79)	<.001* <.001*	1.55 1.58
Jiang et al., 2022	Supportive therapy/Health education	11.1 (1)	0 (0)	PTSD symptoms (PCL-5)	NR	NR	6-mon	33.75 (6.92)	46.11 (8.14)	.004*	1.63
Zolfa et al., 2023	WET/Assessment only	0 (0)	0 (0)	PTSD symptoms (PCL-5)	56.52 (4.68)	55.83 (3.69)	PT 3-mon	34.09 (4.14) 32.30 (2.74)	52.78 (4.92) 52.43 (4.68)	<.001* <.001*	4.11 5.25
Jiang et al., 2023	TMS and TMS + BE/Sham TMS	TMS + BE NR (2) TMS NR (2)	Sham TMS NR (1)	PTSD symptoms (IES-R)	TMS + BE 50.4 (8.5) TMS 49.0 (10.5)	Sham TMS 47.9 (8.6)	3-mon	NR NR	NR	.001* .01*	NA NA

Note. CBT: Cognitive Behavioral Therapy; EMDR: Eye Movement Desensitization and Reprocessing; IES-R: Impact of Event Scale-Revised; NA: not applicable; NR: not reported; PCL-5: PTSD Checklist for DSM-5; PCL-C: PTSD Checklist-Civilian Version; PE: Prolonged Exposure; PSS-I: PTSD Symptom Scale – Interview Version; PT: posttreatment; SD: standard deviation; TMS: repetitive transcranial magnetic stimulation; TMS + BE: repetitive transcranial magnetic stimulation plus brief exposure; WET: Written Exposure Therapy

a *p*-values were calculated for this review based on reported values using *t*-tests (two-tailed, assuming equal variances) comparing posttreatment and follow-up means for treatment and comparison groups.

b Cohen’s d estimates were calculated for this systematic review when available information was reported. Positive values indicate that the treatment condition was favoured over the comparison condition.

## Data Availability

Data were extracted and managed through the Systematic Review Data Depository (SRDR) and are available via SRDR.

## References

[R1] Abbey G, Thompson SB, Hickish T, & Heathcote D. (2015). A meta-analysis of prevalence rates and moderating factors for cancer-related post-traumatic stress disorder. Psychooncology, 24(4), 371–381. 10.1002/pon.3654 25146298 PMC4409098

[R2] Agarwal S, Presciutti A, Cornelius T, Birk J, Roh DJ, Park S, Claassen J, Elkind MSV, & Edmondson D. (2019). Cardiac arrest and subsequent hospitalization–induced posttraumatic stress is associated with 1-year risk of major adverse cardiovascular events and all-cause mortality. Critical Care Medicine, 47(6), e502–e505. 10.1097/CCM.0000000000003713 30889030 PMC6522295

[R3] Al Amer FM, & Lin L. (2021). Empirical assessment of prediction intervals in Cochrane meta-analyses. European Journal of Clinical Investigation, 51(7), e13524. 10.1111/eci.13524 PMC9555293 33595098

[R4] American Psychiatric Association. (1994). Diagnostic and statistical manual of mental disorders (4th ed.).

[R5] American Psychiatric Association. (2013). Diagnostic and statistical manual of mental disorders (5th ed.).

[R6] Arabia E, Manca ML, & Solomon RM (2011). EMDR for survivors of life-threatening cardiac events: Results of a pilot study. Journal of EMDR Practice and Research, 5(1), 2–13.

[R7] Archer J, Bower P, Gilbody S, Lovell K, Richards D, Gask L, Dickens C, & Coventry P. (2012). Collaborative care for depression and anxiety problems. Cochrane Database of Systematic Reviews, 10. 10.1002/14651858.CD006525.pub2 PMC11627142 23076925

[R8] Balduzzi S, Rücker G, & Schwarzer G. (2019). How to perform a meta-analysis with R: A practical tutorial. Evidence Based Mental Health, 22(4), 153–160. 10.1136/ebmental-2019-300117 31563865 PMC10231495

[R9] Benjet C, Bromet E, Karam EG, Kessler RC, McLaughlin KA, Ruscio AM, Shahly V, Stein DJ, Petukhova M, Hill E, Alonso J, Atwoli L, Bunting B, Bruffaerts R, Caldas-de-Almeida JM, de Girolamo G, Florescu S, Gureje O, Huang Y, … Koenen KC (2016). The epidemiology of traumatic event exposure worldwide: Results from the world mental health survey consortium. Psychological Medicine, 46(2), 327–343. 10.1017/S0033291715001981 26511595 PMC4869975

[R10] Blount AE (1998). Integrated primary care: The future of medical and mental health collaboration. WW Norton & Company.

[R11] Capezzani L, Ostacoli L, Cavallo M, Carletto S, Fernandez I, & Solomon R. (2013). EMDR and CBT for cancer patients: Comparative study of effects on PTSD, anxiety, and depression. Journal of EMDR Practice and Research, 7(3), 134–143.

[R12] Carletto S, Borghi M, Bertino G, Oliva F, Cavallo M, Hofmann A, Zennaro A, Malucchi S, & Ostacoli L. (2016). Treating post-traumatic stress disorder in patients with multiple sclerosis: A randomized controlled trial comparing the efficacy of eye movement desensitization and reprocessing and relaxation therapy. Frontiers in Psychology, 7, 526. 10.3389/fpsyg.2016.00526 27148134 PMC4838623

[R13] Chang YC, Tseng TA, Lin GM, Hu WY, Wang CK, & Chang YM (2023). Immediate impact of mindfulness-based cognitive therapy (MBCT) among women with breast cancer: A systematic review and meta-analysis. BMC Women’s Health, 23(1), 331. 10.1186/s12905-023-02486-x 37349700 PMC10288664

[R14] Cordova MJ, Riba MB, & Spiegel D. (2017). Post-traumatic stress disorder and cancer. The Lancet Psychiatry, 4(4), 330–338. 10.1016/S2215-0366(17)30014-7 28109647 PMC5676567

[R15] DuHamel KN, Mosher CE, Winkel G, Labay LE, Rini C, Meschian YM, Austin J, Greene PB, Lawsin CR, Rusiewicz A, Grosskreutz CL, Isola L, Moskowitz CH, Papadopoulos EB, Rowley S, Scigliano E, Burkhalter JE, Hurley KE, Bollinger AR, & Redd WH (2010). Randomized clinical trial of telephone-administered cognitive-behavioral therapy to reduce post-traumatic stress disorder and distress symptoms after hematopoietic stem-cell transplantation. Journal of Clinical Oncology, 28(23), 3754–3761. 10.1200/JCO.2009.26.8722 20625129 PMC2917309

[R16] Edmondson D. (2014). An enduring somatic threat model of posttraumatic stress disorder due to acute life-threatening medical events. Social and Personality Psychology Compass, 8(3), 118–134. 10.1111/spc3.12089 24920956 PMC4048720

[R17] Edmondson D, Richardson S, Falzon L, Davidson KW, Mills MA, & Neria Y. (2012). Posttraumatic stress disorder prevalence and risk of recurrence in acute coronary syndrome patients: A meta-analytic review. PLoS ONE, 7(6), e38915. 10.1371/journal.pone.0038915 PMC3380054 22745687

[R18] Edmondson D, Richardson S, Fausett JK, Falzon L, Howard VJ, & Kronish IM (2013). Prevalence of PTSD in survivors of stroke and transient ischemic attack: A meta-analytic review. PLoS ONE, 8(6), e66435. 10.1371/journal.pone.0066435 PMC3686746 23840467

[R19] Edmondson D, & von Känel R. (2017). Post-traumatic stress disorder and cardiovascular disease. The Lancet Psychiatry, 4(4), 320–329. 10.1016/S2215-0366(16)30377-7 28109646 PMC5499153

[R20] Engel CC, Jaycox LH, Freed MC, Bray RM, Brambilla D, Zatzick D, Litz B, Tanielian T, Novak LA, Lane ME, Belsher BE, Olmsted KLR, Evatt DP, Vandermaas-Peeler R, Unützer J, & Katon WJ (2016). Centrally assisted collaborative telecare for posttraumatic stress disorder and depression among military personnel attending primary care: A randomized clinical trial. JAMA Internal Medicine, 176(7), 948–956. 10.1001/jamainternmed.2016.2402 27294447

[R21] Farris SG, Derby L, & Kibbey MM (2025). Getting comfortable with physical discomfort: A scoping review of interoceptive exposure in physical and mental health conditions. Psychological Bulletin, 151(2), 131–191. 10.1037/bul0000464 40014537 PMC11905771

[R22] Farris SG, Kibbey MM, Derby L, Keller B, Hoyt D, Brinkman HR, Alderman BL, & Leyro TM (2024). Tailoring interoceptive exposure for patients with medical comorbidities. Cognitive and Behavioral Practice, (Advance online publication).

[R23] Ford J, Rosman L, Wuensch KL, Irvine J, & Sears SF (2016). Cognitive–behavioral treatment of posttraumatic stress in patients with implantable cardioverter defibrillators: Results from a randomized controlled trial. Journal of Traumatic Stress, 29(4), 388–392. 10.1002/jts.22111 27415850

[R24] Fortney JC, Pyne JM, Kimbrell TA, Hudson TJ, Robinson DE, Schneider R, Moore WM, Custer PJ, Grubbs KM, & Schnurr PP (2015). Telemedicine-based collaborative care for posttraumatic stress disorder: A randomized clinical trial. JAMA Psychiatry, 72(1), 58–67. 10.1001/jamapsychiatry.2014.1575 25409287

[R25] Haerizadeh M, Sumner JA, Birk JL, Gonzalez C, Heyman-Kantor R, Falzon L, Gershengoren L, Shapiro P, & Kronish IM (2020). Interventions for posttraumatic stress disorder symptoms induced by medical events: A systematic review. Journal of Psychosomatic Research, 129, 109908. 10.1016/j.jpsychores.2019.109908 PMC7580195 31884302

[R26] Hamblen JL, Norman SB, Sonis JH, Phelps AJ, Bisson JI, Nunes VD, Megnin-Viggars O, Forbes D, Riggs DS, & Schnurr PP (2019). A guide to guidelines for the treatment of posttraumatic stress disorder in adults: An update. Psychotherapy, 56(3), 359–373. 10.1037/pst0000231 31282712

[R27] Higgins JP, Thompson SG, Deeks JJ, & Altman DG (2003). Measuring inconsistency in meta-analyses. BMJ, 327(7414), 557–560. 10.1136/bmj.327.7414.557 12958120 PMC192859

[R28] Imel ZE, Laska K, Jakupcak M, & Simpson TL (2013). Meta-analysis of dropout in treatments for posttraumatic stress disorder. Journal of Consulting and Clinical Psychology, 81(3), 394–404. 10.1037/a0031474 23339535 PMC3893277

[R29] Irvine J, Firestone J, Ong L, Cribbie R, Dorian P, Harris L, Ritvo P, Katz J, Newman D, Cameron D, Johnson S, Bilanovic A, Hill A, O’Donnell S, & Sears S. (2011). A randomized controlled trial of cognitive behavior therapy tailored to psychological adaptation to an implantable cardioverter defibrillator. Psychosomatic Medicine, 73(3), 226–233. 10.1097/PSY.0b013e31820afc63 21321256

[R30] Jarero I, Givaudan M, & Osorio A. (2018). Randomized controlled trial on the provision of the EMDR integrative group treatment protocol adapted for ongoing traumatic stress to female patients with cancer-related posttraumatic stress disorder symptoms. Journal of EMDR Practice and Research, 12(3), 94–104.

[R31] Jiang C, Li Z, Du C, Zhang X, Chen Z, Luo G, Wu X, Wang J, Cai Y, Zhao G, & Bai H. (2022). Supportive psychological therapy can effectively treat post-stroke post-traumatic stress disorder at the early stage. Frontiers in Neuroscience, 16, 1007571. 10.3389/fnins.2022.1007571 PMC9583431 36278005

[R32] Jiang C, Li Z, Wang J, Liu L, Luo G, & Zheng X. (2023). Effectiveness of repetitive transcranial magnetic stimulation combined with a brief exposure procedure for post-stroke posttraumatic stress disorder. Journal of Affective Disorders, 326, 89–95. 10.1016/j.jad.2023.01.096 36717030

[R33] McBain S, & Cordova MJ (2024). Medical traumatic stress: Integrating evidence-based clinical applications from health and trauma psychology. Journal of Traumatic Stress, 37(5), 761–767. 10.1002/jts.23075 38970812

[R34] Meinhausen C, Prather AA, & Sumner JA (2022). Posttraumatic stress disorder (PTSD), sleep, and cardiovascular disease risk: A mechanism-focused narrative review. Health Psychology, 41(10), 663–673. 10.1037/hea0001143 35007121 PMC9271141

[R35] Meredith LS, Eisenman DP, Han B, Green BL, Kaltman S, Wong EC, Sorbero M, Vaughan C, Cassells A, Zatzick D, Diaz C, Hickey S, Kurz JR, & Tobin JN (2016). Impact of collaborative care for underserved patients with PTSD in primary care: A randomized controlled trial. Journal of General Internal Medicine, 31(5), 509–517. 10.1007/s11606-016-3588-3 26850413 PMC4835392

[R36] Meredith LS, Wong EC, Marx BP, Han B, Korn AR, Tobin JN, Cassells A, Williamson S, Franco M, Overa CC, Holder T, Lin TJ, & Sloan DM (2024). Design of a hybrid implementation effectiveness cluster randomized controlled trial of delivering written exposure therapy for PTSD in underserved primary care settings. Contemporary Clinical Trials, 138, 107435. 10.1016/j.cct.2024.107435 PMC11146292 38211725

[R37] Mokdad AH, Ballestros K, Echko M, Glenn S, Olsen HE, Mullany E, Lee A, Khan AR, Ahmadi A, Ferrari AJ, Kasaeian A, Werdecker A, Carter A, Zipkin B, Sartorius B, Serdar B, Sykes BL, Troeger C, Fitzmaurice C, … Murray CJL (2018). The state of US health, 1990–2016: Burden of diseases, injuries, and risk factors among US states. JAMA, 319(14), 1444–1472. 10.1001/jama.2018.0158 29634829 PMC5933332

[R38] Pacella ML, Armelie A, Boarts J, Wagner G, Jones T, Feeny N, & Delahanty DL (2012). The impact of prolonged exposure on PTSD symptoms and associated psychopathology in people living with HIV: A randomized test of concept. AIDS and Behavior, 16(5), 1327–1340. 10.1007/s10461-011-0076-y 22012149 PMC4391814

[R39] Page MJ, McKenzie JE, Bossuyt PM, Boutron I, Hoffmann TC, Mulrow CD, Shamseer L, Tetzlaff JM, Akl EA, Brennan SE, Chou R, Glanville J, Grimshaw JM, Hróbjartsson A, Lalu MM, Li T, Loder EW, Mayo-Wilson E, McDonald S, … Moher D. (2021). The PRISMA 2020 statement: An updated guideline for reporting systematic reviews. BMJ, 372, n71. 10.1136/bmj.n71 33782057 PMC8005924

[R40] Pole N. (2007). The psychophysiology of posttraumatic stress disorder: A meta-analysis. Psychological Bulletin, 133(5), 725–746. 10.1037/0033-2909.133.5.725 17723027

[R41] Presciutti A, Shaffer J, Sumner JA, Elkind MS, Roh DJ, Park S, Claassen J, Edmondson D, & Agarwal S. (2020). Hyperarousal symptoms in survivors of cardiac arrest are associated with 13 month risk of major adverse cardiovascular events and all-cause mortality. Annals of Behavioral Medicine, 54(6), 413–422. 10.1093/abm/kaz058 32043140 PMC7246258

[R42] Rauch SAM, Cigrang J, Austern D, & Evans A. (2017). Expanding the reach of effective PTSD treatment into primary care: Prolonged exposure for primary care. Focus, 15(4), 406–410. 10.1176/appi.focus.20170021 31975871 PMC6519517

[R43] Rauch SAM, Venners MR, Ragin C, Ruhe G, Lamp KE, Burton M, Pomerantz A, Bernardy N, Schnurr PP, Hamblen JL, Possemato K, Sripada R, Wray LO, Dollar K, Wade M, Astin MC, & Cigrang JA (2023). Treatment of posttraumatic stress disorder with prolonged exposure for primary care (PE-PC): Effectiveness and patient and therapist factors related to symptom change and retention. Psychological Services, 20(4), 745–755. 10.1037/ser0000783 37326566 PMC10721715

[R44] Robinson P, O’Donohue WT, Byrd MR, Cummings NA, & Henderson DA (2005). Behavioral integrative care: Treatments that work in the primary care setting. New York: Brunner-Routledge.

[R45] Schnurr PP (2017). Focusing on trauma-focused psychotherapy for posttraumatic stress disorder. Current Opinion in Psychology, 14, 56–60. 10.1016/j.copsyc.2016.11.005 28813321

[R46] Schnurr PP, Friedman MJ, Oxman TE, Dietrich AJ, Smith MW, Shiner B, Forshay E, Gui J, & Thurston V. (2013). RESPECT-PTSD: Re-engineering systems for the primary care treatment of PTSD, a randomized controlled trial. Journal of General Internal Medicine, 28(1), 32–40. 10.1007/s11606-012-2166-6 22865017 PMC3539037

[R47] Shemesh E, Annunziato RA, Weatherley BD, Cotter G, Feaganes JR, Santra M, Yehuda R, & Rubinstein D. (2011). A randomized controlled trial of the safety and promise of cognitive-behavioral therapy using imaginal exposure in patients with posttraumatic stress disorder resulting from cardiovascular illness. The Journal of Clinical Psychiatry, 72(2), 168–174. 10.4088/JCP.09m05116blu 20441725

[R48] Sommer JL, Mota N, Edmondson D, & El-Gabalawy R. (2018). Comorbidity in illness-induced posttraumatic stress disorder versus posttraumatic stress disorder due to external events in a nationally representative study. General Hospital Psychiatry, 53, 88–94. 10.1016/j.genhosppsych.2018.02.004 29776731

[R49] Sterne JAC, Savović J, Page MJ, Elbers RG, Blencowe NS, Boutron I, Cates CJ, Cheng H-Y, Corbett MS, Eldridge SM, Emberson JR, Hernán MA, Hopewell S, Hróbjartsson A, Junqueira DR, Jüni P, Kirkham JJ, Lasserson T, Li T, … Higgins JPT (2019). Rob 2: A revised tool for assessing risk of bias in randomised trials. BMJ, 366, l4898. 10.1136/bmj.l4898 31462531

[R50] Sumner JA, Cleveland S, Chen T, & Gradus JL (2023). Psychological and biological mechanisms linking trauma with cardiovascular disease risk. Translational Psychiatry, 13(1), 25. 10.1038/s41398-023-02330-8 36707505 PMC9883529

[R51] Sumner JA, & Edmondson D. (2018). Refining our understanding of PTSD in medical settings. General Hospital Psychiatry, 53, 86–87. 10.1016/j.genhosppsych.2018.05.001 29778269

[R52] US Gov Pr Office. (2023). VA/DoD clinical practice guideline for management of posttraumatic stress disorder and acute stress disorder. 10.1002/jts.23013 38184799

[R53] Vilchinsky N, Ginzburg K, Fait K, & Foa EB (2017). Cardiac-disease-induced PTSD (CDI-PTSD): A systematic review. Clinical Psychology Review, 55, 92–106. 10.1016/j.cpr.2017.04.009 28575815

[R54] Wangelin BC, & Tuerk PW (2015). Taking the pulse of prolonged exposure therapy: Physiological reactivity to trauma imagery as an objective measure of treatment response. Depression and Anxiety, 32(12), 927–934. 10.1002/da.22449 26522237

[R55] Zolfa R, Moradi A, Mahdavi M, Parhoon H, Parhoon K, & Jobson L. (2023). Feasibility and acceptability of written exposure therapy in addressing posttraumatic stress disorder in Iranian patients with breast cancer. Psychooncology, 32(1), 68–76. 10.1002/pon.6037 36116086

