# Supplementary material for: Efficacy of interventions for posttraumatic stress disorder symptoms induced by traumatic medical events: a systematic review
Source: Health Psychol Rev. Author manuscript; Available in PMC 2025 Aug 12. (PMC12341736; doi:10.1080/17437199.2025.2526666)
Supplement: Supp 3 [file NIHMS2095501-supplement-Supp_3.docx]

**Appendix**

**Search Strategy**

**MEDLINE (Ovid)**

1. exp Stress Disorders, Traumatic/

2. (post-traumatic or (post adj traumatic) or posttraumatic).tw.

3. ptsd.tw.

4. or/1-3

5. cardiovascular diseases/

6. (cardiovascular or cvd).tw.

7. exp Myocardial Ischemia/

8. ((myocardial adj infarct$) or mi).tw.

9. (heart adj (attack$ or infarc$ or isch?emia$)).tw.

10. exp Coronary Artery Bypass/

11. (Coronary Artery Bypass or cabg).tw.

12. Angioplasty, Transluminal, Percutaneous Coronary/

13. Percutaneous Transluminal Coronary.tw.

14. (pctci or ptca).tw.

15. (coronary artery disease or cad or acute coronar$ or acs).tw.

16. exp stroke/

17. Stroke$.tw.

18. cerebrovascular.tw.

19. ((brain or vascular or lacunar or venous or cerebral or isch?emic) adj2 (accident$ or infarct$ or event$ or attack$)).tw.

20. (cva or cvas).tw.

21. Ischemic Attack, Transient/

22. (transient adj3 ischemi$).tw.

23. (tia or tias).tw.

24. exp Neoplasms/

25. (cancer$ or neoplasm$ or tumo?r$ or oncolog$).tw.

26. exp Organ Transplantation/

27. ((organ$ or heart$ or kidney$ or lung$ or pancrea$) adj (transplant$ or graft$)).tw.

28. exp HIV Infections/

29. (hiv or Human immunodeficiency virus or aids or Acquired Immune Deficiency Syndrome).tw.

30. exp Asthma/

31. asthma$.tw.

32. acute disease/ or chronic disease/ or critical illness/

33. ((health or medical or physical$ or critical$) adj (ill$ or condition$ or disease$)).tw.

34. (intensive care or icu$).tw.

35. or/5-34

36. 4 and 35

37. randomized controlled trial.pt.

38. controlled clinical trial.pt.

39. randomized.ab.

40. placebo.ab.

41. drug therapy.fs.

42. randomly.ab.

43. trial.ab.

44. groups.ab.

45. or/37-44

46. exp animals/ not humans.sh.

47. 45 not 46

48. 36 and 47

**The Cochrane Library.** All databases are part of *The Cochrane Library*. [www.thecochranelibrary.com](file://///sgbf010.mc.cumc.columbia.edu/DOM_CBCH$/Staff%20folders/Louise/Cochrane/TSC%20Stuff/www.thecochranelibrary.com)

#1 MeSH descriptor: [Stress Disorders, Traumatic] explode all trees

#2 post-traumatic:ti,ab or post next traumatic:ti,ab or posttraumatic:ti,ab

#3 ptsd:ti,ab

#4 #1 or #2 or #3

#5 MeSH descriptor: [Cardiovascular Diseases] this term only

#6 (cardiovascular or cvd):ti,ab

#7 MeSH descriptor: [Myocardial Ischemia] explode all trees

#8 ((myocardial next infarct*) or mi):ti,ab

#9 (heart next (attack* or infarct* or isch?emia*)):ti,ab

#10 MeSH descriptor: [Coronary Artery Bypass] explode all trees

#11 ("Coronary Artery Bypass" or cabg):ti,ab

#12 MeSH descriptor: [Angioplasty, Balloon, Coronary] this term only

#13 "Percutaneous Transluminal Coronary":ti,ab

#14 (pctci or ptca):ti,ab

#15 ("coronary artery disease" or cad or "acute coronar*" or acs):ti,ab

#16 MeSH descriptor: [Stroke] explode all trees

#17 Stroke*:ti,ab

#18 cerebrovascular:ti,ab

#19 ((brain or vascular or lacunar or venous or cerebral or isch?emic) near/2 (accident* or infarct* or event* or attack*)):ti,ab

#20 (cva or cvas):ti,ab

#21 MeSH descriptor: [Ischemic Attack, Transient] this term only

#22 (transient near/3 ischemi*):ti,ab

#23 (tia or tias):ti,ab

#24 MeSH descriptor: [Neoplasms] explode all trees

#25 (cancer* or neoplasm* or tumo?r* or oncolog*) .ti,ab

#26 MeSH descriptor: [Organ Transplantation] explode all trees

#27 ((organ* or heart* or kidney* or lung* or pancrea*) next (transplant* or graft*)):ti,ab

#28 MeSH descriptor: [HIV Infections] explode all trees

#29 (hiv or "Human immunodeficiency virus" or aids or "Acquired Immune Deficiency Syndrome"):ti,ab

#30 MeSH descriptor: [Asthma] explode all trees

#31 asthma*:ti,ab

#32 MeSH descriptor: [Acute Disease] this term only

#33 MeSH descriptor: [Chronic Disease] this term only

#34 MeSH descriptor: [Critical Illness] this term only

#35 ((health or medical or physical* or critical*) next (ill* or condition* or disease*)):ti,ab

#36 ("intensive care" or icu*):ti,ab

#37 #5 or #6 or #7 or #8 or #9 or #10 or #11 or #12 or #13 or #14 or #15 or #16 or #17 or #18 or #19 or #20 or #21 or #22 or #23 or #24 or #25 or #26 or #27 or #28 or #29 or #30 or #31 or #32 or #33 or #34 or #35 or #36

#38 #4 and #37

**EMBASE (EMBASE.com)**

#44. #39 AND #43 AND [humans]/lim AND [embase]/lim

#43. #40 OR #41 OR #42

#42. 'health care quality'/exp

#41. 'clinical trial' OR 'clinical trials'

#40. random*:ab,ti

#39. #4 AND #38

#38. #5 OR #6 OR #7 OR #8 OR #9 OR #10 OR #11 OR #12 OR #13 OR #14 OR #15 OR #16 OR #17 OR #18 OR #19 OR #20 OR #21 OR #22 OR #23 OR #24 OR #26 OR #27 OR #28 OR #29 OR #30 OR #31 OR #32 OR #33 OR #34 OR #35 OR #36 OR #37

#37. 'intensive care':ab,ti OR icu*:ab,ti

#36. ((health OR medical OR physical* OR critical*) NEAR/2 (ill* OR condition* OR disease*)):ab,ti

#35. 'critical illness'/de

#34. 'chronic disease'/de

#33. 'acute disease'/de

#32. asthma*:ab,ti

#31. 'asthma'/exp

#30. hiv:ab,ti OR 'human immunodeficiency virus':ab,ti OR aids:ab,ti OR 'acquired immune deficiency syndrome':ab,ti

#29. 'human immunodeficiency virus infection'/exp

#28. organ*:ab,ti OR heart*:ab,ti OR kidney*:ab,ti OR lung*:ab,ti OR pancrea*:ab,ti AND next:ab,ti AND (transplant*:ab,ti OR graft*:ab,ti)

#27. 'organ transplantation'/exp

#26. cancer*:ab,ti OR neoplasm*:ab,ti OR tumour*:ab,ti OR tumor*:ab,ti OR oncolog*:ab,ti

#24. 'neoplasm'/exp

#23. tia:ab,ti OR tias:ab,ti

#22. (transient NEAR/3 ischemi*):ab,ti

#21. 'transient ischemic attack'/de

#20. cva:ab,ti OR cvas:ab,ti

#19. ((brain OR vascular OR lacunar OR venous OR cerebral OR isch?emic) NEAR/2 (accident* OR

infarct* OR event* OR attack*)):ab,ti

#18. 'cerebrovascular':ab,ti

#17. stroke*:ab,ti

#16. 'cerebrovascular accident'/exp

#15. 'coronary artery disease':ab,ti OR cad:ab,ti OR 'acute coronary':ab,ti OR 'acute coronoraries':ab,ti OR acs:ab,ti

#14. pctci:ab,ti OR ptca:ab,ti

#13. 'percutaneous transluminal coronary':ab,ti

#12. 'percutaneous transluminal angioplasty'/de

#11. 'coronary artery bypass':ab,ti OR cabg:ab,ti

#10. 'coronary artery bypass graft'/de

#9. (heart NEAR/2 (attack* OR infarct* OR isch?emia*)):ab,ti

#8. 'myocardial infarct':ab,ti AND 'myocardial infarcts':ab,ti OR 'myocardial infarction':ab,ti

OR 'myocardial infarctions':ab,ti OR mi:ab,ti

#7. 'heart muscle ischemia'/exp

#6. cardiovascular:ab,ti OR cvd:ab,ti

#5. 'cardiovascular disease'/de

#4. #1 OR #2 OR #3

#3. ptsd:ab,ti

#2. 'post traumatic':ab,ti OR posttraumatic:ab,ti

#1. 'posttraumatic stress disorder'/de

**PsycINFO (Ovid)**

1. posttraumatic stress disorder/

2. (post-traumatic or (post adj traumatic) or posttraumatic).tw.

3. ptsd.tw.

4. or/1-3

5. cardiovascular disorders/

6. (cardiovascular or cvd).tw.

7. myocardial infarctions/

8. ((myocardial adj infarct$) or mi).tw.

9. (heart adj (attack$ or infarct$ or isch?emia$)).tw.

10. heart surgery/

11. (Coronary Artery Bypass or cabg).tw.

12. Percutaneous Transluminal Coronary.tw.

13. (pctci or ptca).tw.

14. (coronary artery disease or cad or acute coronar$ or acs).tw.

15. cerebrovascular accidents/

16. Stroke$.tw.

17. cerebrovascular.tw.

18. ((brain or vascular or lacunar or venous or cerebral or isch?emic) adj2 (accident$ or infarct$ or event$ or attack$)).tw.

19. (cva or cvas).tw.

20. exp cerebral ischemia/

21. (transient adj3 ischemi$).tw.

22. (tia or tias).tw.

23. exp Neoplasms/

24. (cancer$ or neoplasm$ or tumo?r$ or oncolog$).tw.

25. organ transplantation/

26. ((organ$ or heart$ or kidney$ or lung$ or pancrea$) adj (transplant$ or graft$)).tw.

27. exp hiv/

28. (hiv or Human immunodeficiency virus or aids or Acquired Immune Deficiency Syndrome).tw.

29. asthma/

30. asthma$.tw.

31. physical disorders/

32. chronic illness/

33. intensive care/

34. ((health or medical or physical$ or critical$) adj (ill$ or condition$ or disease$)).tw.

35. (intensive care or icu$).tw.

36. or/5-35

37. 4 and 36

38. limit 37 to (human and "2000 treatment outcome/clinical trial")

**CINAHL (EBSCOhost)**

S1 (MH "Stress Disorders, Post-Traumatic+")

S2 TI ( post-traumatic OR posttraumatic OR post traumatic ) OR AB ( post-traumatic OR posttraumatic OR post traumatic )

S3 TI ptsd OR AB ptsd

S4 S1 OR S2 OR S3

S5 (MH "Cardiovascular Diseases")

S6 TI ( cardiovascular OR cvd ) OR AB ( cardiovascular OR cvd )

S7 (MH "Myocardial Ischemia+")

S8 TI ( myocardial infarct* OR mi ) OR AB ( myocardial infarct* OR mi )

S9 TI heart attack* OR AB heart attack* OR TI heart infarct* OR AB heart infarct* OR TI heart isch?emia* OR AB heart isch?emia*

S10 (MH "Coronary Artery Bypass+")

S11 TI Coronary Artery Bypass or TI cabg OR AB Coronary Artery Bypass or AB cabg

S12 (MH "Angioplasty, Transluminal, Percutaneous Coronary")

S13 TI Percutaneous Transluminal Coronary OR AB Percutaneous Transluminal Coronary

S14 TI pctci OR AB ptca OR TI pctci OR AB ptca

S15 TI coronary artery disease OR AB coronary artery disease OR TI cad OR AB cad OR TI acute coronar* OR AB acute coronar* OR TI acs OR AB acs

S16 (MH "Stroke+")

S17 TI Stroke* OR AB Stroke*

S18 TI cerebrovascular OR AB cerebrovascular

S19 TI ( ((brain OR vascular OR lacunar OR venous OR cerebral OR isch?emic) N2 (accident* OR infarct* OR event* OR attack*)) ) OR AB ( ((brain OR vascular OR lacunar OR venous OR cerebral OR isch?emic) N2 (accident* OR infarct* OR event* OR attack*)) )

S20 TI ( cva OR cvas ) OR AB ( cva OR cvas )

S21 (MH "Cerebral Ischemia, Transient")

S22 TI transient N3 ischemi* OR AB transient N3 ischemi*

S23 TI ( tia OR tias ) OR AB ( tia OR tias )

S24 (MH "Neoplasms+")

S25 TI ( cancer* OR neoplasm* OR tumo?r* OR oncolog* ) OR AB ( cancer* OR neoplasm* OR tumo?r* OR oncolog* )

S26 (MH "Organ Transplantation+")

S27 TI ( ((organ* OR heart* OR kidney* OR lung* OR pancrea*) N2 (transplant* OR graft*)) ) OR AB ( ((organ* OR heart* OR kidney* OR lung* OR pancrea*) N2 (transplant* OR graft*)) )

S28 (MH "HIV Infections+")

S29 TI hiv OR AB hiv OR TI Human immunodeficiency virus OR AB Human immunodeficiency virus OR TI aids OR AB aids OR TI Acquired Immune Deficiency Syndrome OR AB Acquired Immune Deficiency Syndrome

S30 (MH "Asthma+")

S31 TI Asthma* OR AB Asthma*

S32 (MH "Acute Disease")

S33 (MH "Chronic Disease")

S34 (MH "Critical Illness")

S35 TI ( ((health OR medical OR physical* OR critical*) N2 (ill* OR condition* OR disease*)) ) OR AB ( ((health OR medical OR physical* OR critical*) N2 (ill* OR condition* OR disease*)) )

S36 TI ( intensive care OR icu* ) OR AB ( intensive care OR icu* )

S37 S5 OR S6 OR S7 OR S8 OR S9 OR S10 OR S11 OR S12 OR S13 OR S14 OR S15 OR S16 OR S17 OR S18 OR S19 OR S20 OR S21 OR S22 OR S23 OR S24 OR S25 OR S26 OR S27 OR S28 OR S29 OR S30 OR S31 OR S32 OR S33 OR S34 OR S35 OR S36

S38 S4 AND S37 Limiters - Exclude MEDLINE records; Human

**PTSD Pubs (formerly PILOTS (ProQuest))**

Set#: S1

Searched for: ti(cardiovascular OR cvd) OR ab(cardiovascular OR cvd)

Set#: S2

Searched for: ti("myocardial infarct*" OR mi) OR ab("myocardial infarct*" OR mi)

Set#: S3

Searched for: ti("heart attack*") OR ab("heart attack*") OR ti("heart infarct*") OR ab("heart infarct*") OR ti("heart isch?emia*") OR ab("heart isch?emia*")

Set#: S4

Searched for: ti("Coronary Artery Bypass") OR ab("Coronary Artery Bypass") OR ti(cabg) OR ab(cabg)

Set#: S5

Searched for: SU.EXACT.EXPLODE("Cardiovascular Diseases")

Set#: S6

Searched for: ti("Percutaneous Transluminal Coronary") OR ab("Percutaneous Transluminal Coronary")

Set#: S7

Searched for: ti(pctci ) OR ab(pctci ) OR ti(pcta) AND ab(pcta)

Set#: S9

Searched for: ti("coronary artery disease") OR ab("coronary artery disease") OR ti(cad) AND ab(cad) OR ti("acute coronar*") OR ab("acute coronar*") OR ti(acs) OR ab(acs)

Set#: S10

Searched for: ti(stroke*) OR ab(stroke*)

Set#: S11

Searched for: ti( cerebrovascular ) OR ab( cerebrovascular )

Set#: S12

Searched for: ((brain OR vascular OR lacunar OR venous OR cerebral OR isch?emic) Near/2 (accident* OR infarct* OR event* OR attack*))

Set#: S13

Searched for: ((brain OR vascular OR lacunar OR venous OR cerebral OR isch?emic) Near/2 (accident* OR infarct* OR event* OR attack*))

Set#: S14

Searched for: ti(cva) OR ab(cva) OR ti(cvas) OR ab(cvas)

Set#: S15

Searched for: ti(transient Near/3 ischemi* ) OR ab(transient Near/3 ischemi* )

Set#: S16

Searched for: ti(tia) OR ab(tias) OR ti(tia) OR ab(tias)

Set#: S19

Searched for: SU.EXACT.EXPLODE("Cancer")

Set#: S20

Searched for: ti(cancer*) OR ab(cancer*) OR ti(neoplasm*) OR ab(neoplasm*) OR ti(tumo?r* ) OR ab(tumo?r* ) OR ti(oncolog*) OR ab(oncolog*)

Set#: S20

Searched for: SU.EXACT.EXPLODE("Bone Marrow Transplantation" OR "Organ Transplantation")

Set#: S21

Searched for: ti((organ* OR heart* OR kidney* OR lung* OR pancrea*) Near/2 (transplant* OR graft*))

Set#: S22

Searched for: ab((organ* OR heart* OR kidney* OR lung* OR pancrea*) Near/2 (transplant* OR graft*))

Set#: S23

Searched for: SU.EXACT.EXPLODE("AIDS")

Set#: S24

Searched for: ti(hiv) OR ab(hiv) OR ti("Human immunodeficiency virus") OR ab("Human immunodeficiency virus") OR ti(aids) OR ab(aids) OR ti("Acquired Immune Deficiency Syndrome") OR ab("Acquired Immune Deficiency Syndrome")

Set#: S25

Searched for: SU.EXACT.EXPLODE("Respiratory Diseases")

Set#: S26

Searched for: ti(asthma*) OR ab(asthma*)

Set#: S27

Searched for: SU.EXACT("Chronic Diseases")

Set#: S28

Searched for: ti((health OR medical OR physical* OR critical*) Near/2 (ill* OR condition* OR disease*))

Set#: S29

Searched for: ab((health OR medical OR physical* OR critical*) Near/2 (ill* OR condition* OR disease*))

Set#: S30

Searched for: ti("intensive care") OR ab("intensive care") OR ti(icu*) OR ab(icu*)

Set#: S31

1 OR 2 OR 3 OR 4 OR 5 OR 6 OR 7 OR 8 OR 9 OR 10 OR 11 OR 12 OR 13 OR 14 OR 15 OR 16 OR 17 OR 18 OR 19 OR 20 OR 21 OR 22 OR 23 OR 24 OR 25 OR 26 OR 27 OR 28 OR 29 OR 30

Set#: S32

Searched for: SU.EXACT.EXPLODE("(Stress Disorder Symptoms)" OR "Anniversary Reactions" OR "Arousal" OR "Auditory Hallucinations" OR "Avoidance" OR "Comorbidity" OR "Complex PTSD" OR "Dissociative Symptoms" OR "Electrodermal Activity" OR "Emotional Numbing" OR "Hallucinations" OR "Hypervigilance" OR "Hypnagogic Hallucinations" OR "Intrusive Thoughts" OR "Olfactory Hallucinations" OR "PTSD" OR "PTSD (DSM-5)" OR "PTSD (DSM-III)" OR "PTSD (DSM-III-R)" OR "PTSD (DSM-IV)" OR "PTSD (ICD-10)" OR "PTSD (ICD-11)" OR "PTSD (ICD-9)" OR "Reexperiencing" OR "Somatic Hallucinations" OR "Startle Reflex" OR "Tactile Hallucinations" OR "Visual Hallucinations")

Set#: S33

Searched for: ti(post-traumatic OR posttraumatic OR "post traumatic") OR ab(post-traumatic OR posttraumatic OR "post traumatic")

Set#: S34

Searched for: ti(ptsd) OR ab(ptsd)

Set#: S35

32 OR 33 OR 34

Set#: S36

31 AND 35

Set#: S37

Searched for: (SU.EXACT.EXPLODE("Clinical Trial" OR "Randomized Clinical Trial") OR ti(random*) OR ab(random*))
Set#: S38

37 AND 35

**Clinicaltrials.gov**

Completed Studies | Studies With Results | Interventional Studies | "Stress Disorders, Post-Traumatic"

**Google and Google Scholar** (limited to the first 200 results)

“Medically induced PTSD”

PTSD medical

PTSD hospital

PTSD ICU

**PTSD Repository**

Trauma type: Medical/Illness
